# Project Safe Guard–Trauma (PSG-T): Protocol for a randomized controlled trial of lethal means safety counseling to promote secure firearm storage among individuals with PTSD

**DOI:** 10.1016/j.conctc.2025.101549

**Published:** 2025-09-10

**Authors:** Ian H. Stanley, Julia Finn, Kathleen M. Flarity, Mengli Xiao, Rachel L. Johnson, Jaclyn C. Kearns, Natalie L. Wilver, Steven J. Berkowitz, Michael D. Anestis, Marian E. Betz, Joseph A. Simonetti

**Affiliations:** a Department of Emergency Medicine, University of Colorado Anschutz Medical Campus, Aurora, CO, USA; b Center for COMBAT Research, University of Colorado Anschutz Medical Campus, Aurora, CO, USA; c Firearm Injury Prevention Initiative, University of Colorado Anschutz Medical Campus, Aurora, CO, USA; d Marcus Institute for Brain Health, University of Colorado Anschutz Medical Campus, Aurora, CO, USA; e Department of Biostatistics and Informatics, University of Colorado Anschutz Medical Campus, Aurora, CO, USA; f Behavioral Science Division, National Center for PTSD, VA Boston Healthcare System, Boston, MA, USA; g Boston University Chobanian & Avedisian School of Medicine, Boston, MA, USA; h Natalie Wilver, PhD, PLLC, Fairfax, VA, USA; i Department of Psychiatry, University of Colorado Anschutz Medical Campus, Aurora, CO, USA; j New Jersey Gun Violence Research Center, Rutgers University, Piscataway, NJ, USA; k Division of Hospital Medicine, University of Colorado Anschutz Medical Campus, Aurora, CO, USA

**Keywords:** Clinical trial, Suicide, Firearms

## Abstract

Firearm injury is the most common suicide method. When firearms are stored in a non-secure manner (e.g., unlocked, loaded), risk for suicide may be elevated. Accordingly, clinical, public health, and firearm industry stakeholders recommend efforts to promote secure firearm storage, such as lethal means safety counseling (LMSC). One LMSC intervention, Project Safe Guard (PSG), has demonstrated efficacy in prompting use of firearm locking devices in a sample of military service members; however, subsequent analyses show that PSG has diminished efficacy for individuals with elevated symptoms of posttraumatic stress disorder (PTSD). PTSD, characterized in part by hypervigilance to threat, is associated with elevated suicide risk as well as a greater likelihood of storing firearms using less secure methods. In response, our group developed an adaptation of PSG, termed Project Safe Guard-Trauma (PSG-T). This paper describes the design, methodology, and protocol of a randomized controlled trial comparing PSG-T to PSG among adults who screen positive for PTSD related to a victimization trauma (e.g., physical assault, sexual assault, combat) and who do not currently store all their personally owned firearms in a secure manner. PSG and PSG-T will be delivered by licensed clinical psychologists. Assessments will occur at pre-intervention, post-intervention, and 1-, 3-, and 6-month follow-up. The primary objective is to determine the efficacy of PSG-T in prompting greater beliefs and practices regarding secure storage of personal firearms.

## 1 Introduction

Firearm injury is the most common suicide method in the United States [1]. Dozens of case-controlled and ecological studies have shown that access to firearms is associated with increased suicide risk [[2], [3], [4], [5]]. The suicide risk conferred by firearm access persists even after accounting for other suicide risk factors, such as mental illness [[6], [7], [8]]. Among firearm owners, the way in which firearms are stored—for instance, loaded with ammunition and/or unlocked—may also associated with suicide risk [2,5].

To reduce the risk of firearm suicide, multiple stakeholders—spanning clinical, firearm industry, governmental, and non-profit sectors—recommend that firearms be stored securely [[9], [10], [11], [12]]. Secure firearm storage involves a range of possible behaviors, including storing a firearm unloaded, locked, separate from ammunition, and in a secure location [13]. Secure firearm storage can also include voluntarily moving a firearm out of the home for safekeeping with a friend, family member, gun shop, police station, or other community partner [14,15]. A prominent approach to encouraging secure firearm storage is delivery of lethal means safety counseling (LMSC). LMSC refers to guided discussions, often by a clinician, to encourage an at-risk individual to reduce access to firearms and other lethal methods of suicide [13,16].

Project Safe Guard (PSG) is a LMSC intervention delivered through a brief interaction that uses motivational interviewing (MI) principles to encourage behavior change [17]. A randomized controlled trial (RCT) compared the efficacy of PSG, either alone or in combination with the provision of firearm cable locks, in prompting secure storage practices compared to a health counseling control among 232 firearm-owning military service members [17]. At 6-month follow-up, PSG outperformed the control intervention in prompting new use of locking devices (55.0 % vs. 39.0 %; OR = 1.91, 95 % CI = 1.10–3.32), and the combination of PSG and cable lock provision was not superior to either intervention alone. However, a secondary analysis of the PSG RCT found that the intervention-related benefits were attenuated for participants with elevated posttraumatic stress disorder (PTSD) symptoms [18]. This is especially concerning considering research demonstrating that individuals with PTSD are significantly more likely than individuals without PTSD to think about, attempt, and die by suicide [[19], [20], [21], [22]].

**Fig. 1:**
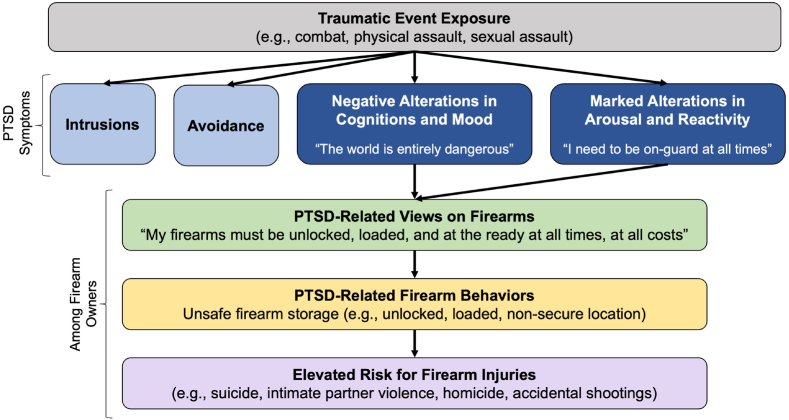
Theoretical model of impact of PTSD symptoms on firearm storage practices.

There are multiple reasons why the efficacy of safety interventions, such as PSG, may have differential impacts on patients with PTSD. PTSD affects safety perceptions and firearm storage practices (Fig. 1) [23]. PTSD is characterized by symptoms that have their onset or worsen following exposure to a traumatic event, persist for at least one month, and cause clinically significant distress or impairment [24]. The core symptoms of PTSD span four primary domains—intrusions, avoidance, negative alterations in cognitions and mood (e.g., beliefs that the world is unsafe), and marked alterations in arousal and reactivity/hyperarousal (e.g., being “on guard” for potential dangers). Individuals with PTSD, versus those without PTSD, report stronger beliefs that firearms protect from victimization [25], and individuals who report protection from others as a primary reason for firearm ownership, versus those who do not, are more likely than others to store their firearms non-securely [23,[26], [27], [28]]. In a sample of 327 firearm-owning military service members, elevated levels of PTSD hyperarousal symptoms were associated with increased odds of storing a firearm loaded and in a non-secure location [29].

Given that greater PTSD symptoms are associated with greater likelihood of non-secure firearm storage practices and existing LMSC interventions, namely PSG, demonstrate diminished efficacy in the setting of elevated PTSD symptoms, LMSC needs to be adapted for use in PTSD populations. The lack of evidence-based approaches for LMSC among individuals with PTSD likely contributes to gaps in care. In one study of 339 clinical psychologists, only 15% reported providing firearm-specific LMSC to their patients with PTSD [30]. In response, we developed an adaptation of PSG, termed Project Safe Guard-Trauma (PSG-T), designed for use with individuals who have PTSD to promote secure firearm storage practices, thereby reducing the risk of firearm-related harms such as suicide. PSG-T extends the MI practices demonstrated to be effective in PSG by exploring and addressing, including with psychoeducation, the potential functional link between trauma exposure and PTSD symptoms with firearm storage practices.

This paper describes the design, methodology, and protocol of an RCT examining the efficacy of PSG-T compared with PSG among firearm-owning individuals who screen positive for PTSD related to a victimization trauma (e.g., physical assault, sexual assault, combat, captivity). The overarching goal of the study is to expand the applicability of LMSC across at-risk populations, including individuals with PTSD for whom existing interventions demonstrate diminished efficacy. Secondary objectives are to evaluate a potential mechanism of change and heterogeneity of treatment effects (HTEs).

## 2 Research aim and hypotheses

This study has two aims and related hypotheses. Aim 1 is to conduct a two-armed RCT to evaluate the efficacy of PSG-T compared with a control (the PSG intervention that does not focus on PTSD) in increasing knowledge about the link between firearm storage practices and suicide risk, intentions to store firearms securely, and secure firearm storage behaviors. Hypothesis 1 is that compared with control, at 1-, 3-, and 6-month follow-up, PSG-T will result in greater (a) adoption of secure firearm storage practices (primary outcome at 6-month follow-up), (b) knowledge about the link between firearm storage practices and suicide risk, and (c) intentions to store firearms securely.

Aim 2 is to evaluate differences in PTSD-related negative cognitions about the world as a potential mechanism of change in PSG-T for increasing knowledge, intentions, and behaviors regarding secure firearm storage practices. Hypothesis 2 is that participants who receive PSG-T will report fewer PTSD-related negative cognitions about the world compared with participants who receive PSG, and these decreases will be associated with increases in (a) adoption of secure firearm storage practices (b) knowledge about the link between firearm storage practices and suicide risk, and (c) intentions to store firearms securely.

## 3 Materials and methods

### 3.1 Study design

**Fig. 2:**
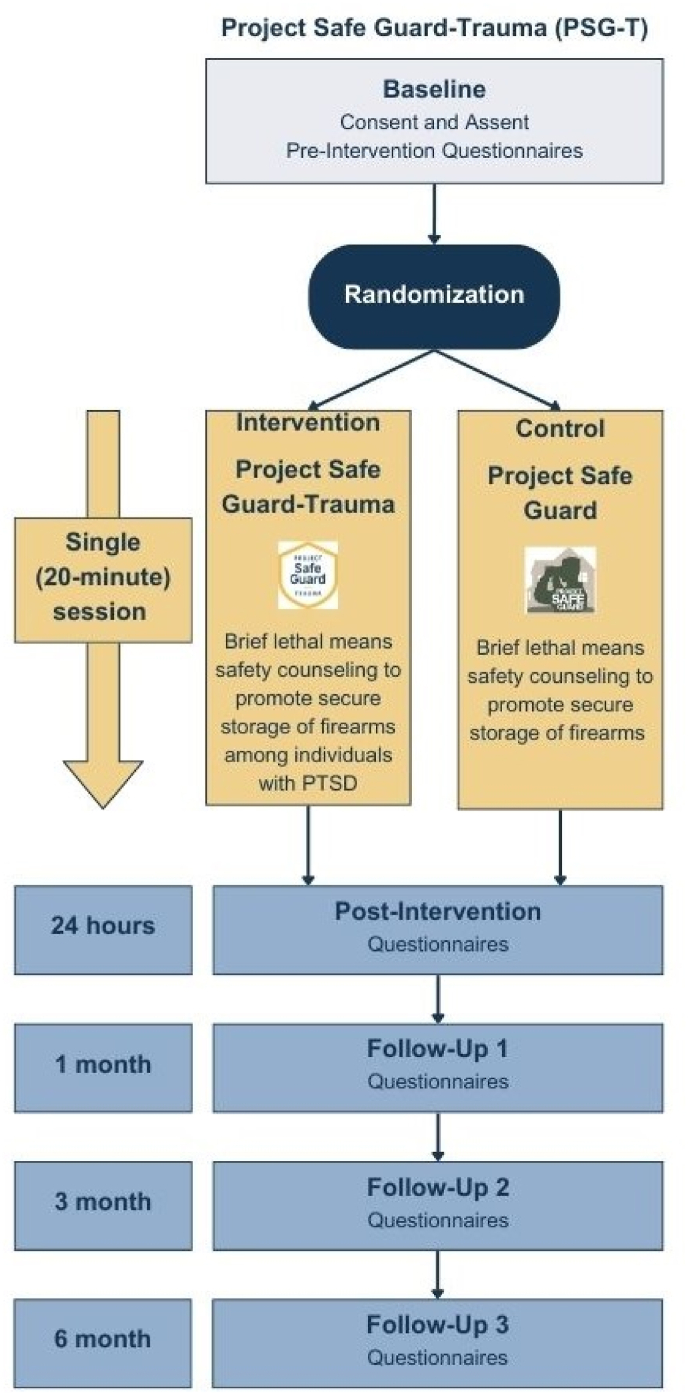
Study design overview.

This study is an RCT examining the efficacy of PSG-T compared with PSG. The overview of the study design is summarized in Fig. 2.

### 3.2 Participants, inclusion and exclusion criteria

Eligible participants will be adults aged 18+ years who screen positive for PTSD on the Primary Care PTSD Screen for DSM-5 (PC-PTSD-5) related to a victimization trauma (e.g., physical assault, sexual assault, military combat) and who do not currently store all their personally owned firearms securely (i.e., unloaded and locked). See Table 1 for inclusion and exclusion criteria.

**Table 1:** Inclusion and exclusion criteria and rationale.

Inclusion Criteria	Rationale
Aged 18 year or older	Population under study
Firearm owner	Population under study
History of one or more victimization traumas per the Life Events Checklist for DSM-5 (LEC-5), defined as having directly experienced physical assault, sexual assault, combat, and/or captivity	Treatment confound
Positive PTSD screen (Primary Care PTSD Screen for DSM-5 [PC-PTSD-5])	Population under study
Willing to provide physical location at time of Zoom sessions	Human subjects concern
Willing to keep Zoom camera on during study sessions	Human subjects concern
Ability to read, write, and speak English	Treatment confound

Exclusion Criteria	Rationale
Currently stores all personal firearms unloaded and locked	Intervention target
Active psychosis or acute mania necessitating clinical intervention	Human subjects concern
Acute thoughts of self- or other-harm necessitating imminent clinical intervention (e.g., hospitalization)	Human subjects concern
Known history of violent or threatening behavior toward hospital staff	Human subjects concern
Unable to provide informed consent	Human subjects concern

#### 3.2.1 Ethical oversight

This study has been approved by the Colorado Multiple Institutional Review Board (COMIRB). The study has also been approved by the US Department of Defense's (DOD's) Office of Human Research Oversight. The trial has been registered at ClinicalTrials.gov (NCT06876740).

### 3.3 Recruitment and informed consent

Participants will be recruited from clinics (e.g., outpatient mental health, primary care) affiliated with the University of Colorado Anschutz Medical Campus. Additionally, physical advertisements (e.g., flyers, brochures) and social media advertisements may be used. Interested participants will be directed to a study eligibility screening questionnaire. Eligible respondents will be contacted to schedule an appointment to describe the study, answer any questions, review the risks and benefits of the study, and ensure the participant understands the research. All study procedures will occur virtually over a HIPAA-compliant instance of Zoom, and informed consent will be obtained digitally through REDCap.

### 3.4 Measures

Participant data will be gathered and stored via self-reported, web-based questionnaires using REDCap. See Table 2 for an overview of the assessments administered and their timelines.

**Table 2:** Study measures by assessment period.

Study Assessments	Pre-Intervention	Post-Intervention	1-, 3-, and-6-Month Follow-Ups
Demographic and Military Service Characteristics	X		
Firearm Ownership and Storage Practices	X	X	X
Intentions to Store Firearms Safely	X	X	X
Firearm-Related Knowledge Questionnaire	X	X	X
Life Events Checklist for DSM-5 (LEC-5)	X		
PTSD Checklist for DSM-5 (PCL-5)	X		X
Posttraumatic Cognitions Inventory (PTCI)	X	X	X
Basic Psychological Needs Satisfaction and Frustration Scale-Single Items (BPNSFS-SI)	X	X	X
Depressive Symptom Index-Suicidality Subscale (DSI-SS)	X		X
Self-Injurious Thoughts and Behaviors Interview-Revised (SITBI-R)	X		X
Client Satisfaction Questionnaire-8 (CSQ-8)		X	X

#### 3.4.1 Demographic characteristics

Participants will provide information about their age, sex, race/ethnicity, marital status, household composition, educational attainment, and military service experiences.

#### 3.4.2 Firearm ownership and storage beliefs and practices

Participants will complete a comprehensive self-report measure of their firearm storage practices, including current use of firearm locking devices (both keyed and biometric devices), reasons for using or not using a locking device, likeliness that participant will encounter a situation that requires use of firearm, and likeliness to adhere to clinician's secure storage recommendations.

The primary outcome will be assessed by asking participants, “Thinking about the firearms in or around your home that you personally own, do you store them using any of the following methods?” Participants will be presented with two methods: “unloaded” and “locked.” For each method, they will be asked to indicate *Yes, all of my firearms*, *Yes, some of my firearms*, or *No, none of my firearms*. We assign a score of 2 if the secure practice applies to all firearms, 1 if it applies to some firearms, and 0 if it applies to none. A higher score corresponds to more secure storage practices. The maximum score, achieved when all firearms are both unloaded and locked, is 4, while the minimum score is 0. Improvement will be defined as a change score >0 between the pre-intervention and 6-month follow-up assessments.

#### 3.4.3 Firearm-related knowledge

Participants will complete a questionnaire about the link between firearm storage practices and suicide risk.

#### 3.4.4 Life Events Checklist for *DSM-5* (LEC-5)

The LEC-5 is a 17-item self-report measure of lifetime exposure to various traumatic events [31]. Participants indicate if each event happened to them, they witnessed it, they learned about it, it was part of their job, not sure, or does not apply.

#### 3.4.5 Primary Care PTSD Screen for DSM-5 (PC-ptsd-5)

Consistent with the VA/DOD Clinical Practice Guideline (CPG) [32], we will use the PC-PTSD-5 to screen individuals at the eligibility determination stage. Participants respond Yes/No to five PTSD symptoms in the past month (score range: 0–5), and scores 4 or greater indicate a positive screen [33]. Individuals will complete the PC-PTSD-5 if they indicate they have experienced a victimization trauma, defined as physical assault, sexual assault, combat, or captivity (cf. LEC-5 items 6–11) [34].

#### 3.4.6 PTSD Checklist for DSM-5 (PCL-5)

The PCL-5 is a 20-item self-report measure to evaluate PTSD symptoms over the past month [35]. Participants indicate how much they were bothered by PTSD symptoms from *not at all* to *extremely* in the past month (score range: 0–80). Consistent with the VA/DOD CPG [32], we will use the PCL-5 to monitor PTSD symptom levels across the study assessment timepoints.

#### 3.4.7 Posttraumatic Cognitions Inventory (PTCI)

The PTCI is a 33-item self-report measure of PTSD-related negative cognitions about the self, world, and self-blame, which can occur following a traumatic event [36]. Participants indicate how much they agree or disagree on a 7-point Likert scale.

#### 3.4.8 Basic Psychological Needs Satisfaction and Frustration Scale-Single Items (BPNSFS-SI)

The BPNSFS-SI is a 3-item self-report measure of self-determination theory constructs of autonomy, relatedness, and competence [37]. Participants rate each item on a 7-point Likert scale (score range: 3–21).

#### 3.4.9 Depressive Symptom Index-Suicidality Subscale (DSI-SS)

The DSI-SS is a 4-item self-report measure of the severity of suicidal ideation over the past 2 weeks [38]. Participants rate each item on a 4-point scale (score range: 0–12).

#### 3.4.10 Self-Injurious Thoughts and Behaviors Interview-Revised (SITBI-R)

The gate questions to each of the self-report SITBI-R modules will be used to evaluate suicidal thoughts and behaviors [39]. We will use the SITBI-R to assess history of these experiences at baseline and between-session experiences at follow-up assessments. Participants respond Yes/No to each item.

#### 3.4.11 Client Satisfaction Questionnaire-8 (CSQ-8)

The CSQ-8 is an 8-item self-report measure that will be used to evaluate participant acceptability of the PSG-T intervention [40]. Participants respond to each item using varying descriptive scales with corresponding values.

### 3.5 Randomization

Participants who meet eligibility criteria will be equally randomized between the two intervention conditions following the pre-intervention assessment. We will use computerized block randomization to allocate participants into either PSG-T or PSG. Block randomization will vary by the number of participants in each block to ensure that the research staff cannot anticipate the next randomization, which mitigates the potential for bias. Biostatisticians will oversee randomization. Neither study clinicians nor data analysts will be blinded.

### 3.6 Intervention conditions

#### 3.6.1 Project Safe Guard-Trauma (PSG-T)

PSG-T is a single-session, brief (20-min) LMSC intervention that promotes secure firearm storage practices among individuals with PTSD. PSG-T promotes secure firearm storage practices through MI [13,41] and by addressing the potential functional link between PTSD symptoms and non-secure storage practices [23]. MI-based LMSC focuses on respecting participants' autonomy, normalizing firearm ownership, and by guiding individuals to identify their values and motivations for secure firearm storage instead of prescribing specific actions. MI-based LMSC uses techniques to develop discrepancies, amplify ambivalence, express empathy, roll with resistance, and support self-efficacy [42]. PSG-T combines MI techniques from PSG [17], with a specific focus on gearing open-ended questions toward exploring the potential for trauma to have impacted one's storage practices and considering how enhancing secure firearm storage may be valuable. PSG-T also includes PTSD and firearm-specific psychoeducation, which focuses on the functional link between PTSD symptoms and firearm storage practices. The PSG-T clinician will work with the participant to generate a written plan for secure firearm storage, based on the participant's stated goals and intentions. A 1-page handout will be provided to participants recapping the points discussed (e.g., firearm-related harms, impact of trauma on firearm storage practices, firearm locking device options, written plan for secure firearm storage, and encouragement to share the document with someone else).

#### 3.6.2 Project Safe Guard (PSG)

PSG is a single-session, brief (20-min) LMSC that promotes secure firearm storage practices using MI principles. The PSG clinician will work with the participant to generate a written plan for secure firearm storage, based on the participant's stated goals and intentions.

#### 3.6.3 Crisis Response Planning (CRP)

Participants across study conditions will also receive crisis response planning (CRP) [43]. CRP is a brief intervention that helps individuals preemptively identify their personal warning signs for a crisis, coping strategies to implement, social supports to activate, and professional services to access. LMSC is not a component of CRP. CRP is associated with a reduced risk for suicide attempts and suicidal ideation, including in the context of PTSD psychotherapy [43,44]. CRP will be delivered in the same session as, and immediately before, PSG-T or PSG. For this trial, the narrative assessment component of CRP will not be used given the population under study is not selected for suicide risk. The decision to include CRP was twofold. First, in standard clinical practice, LMSC is commonly delivered in the context of CRP or safety planning interventions. Second, all participants will have screened positive for PTSD, and although suicidal ideation is not an inclusion criterion, risk of suicide is elevated in those with PTSD. Thus, including CRP as part of the intervention session will provide an added layer of risk mitigation.

### 3.7 Quality control

#### 3.7.1 Training and supervision of study interventionists

All study interventionists will have a master's or doctoral degree in clinical psychology and have experience in treating individuals with PTSD and suicide-related concerns. PSG training involves a 3-h session of didactic lectures from Dr. Michael Anestis, a licensed clinical psychologist and the Principal Investigator (PI) on the initial PSG RCT. PSG-T training involves training in both PSG as well as an additional 1-h session of readings and didactic lectures from Dr. Ian Stanley, a licensed clinical psychologist and the PI of this PSG-T RCT. All study interventionists will also receive guided readings on firearm laws relevant to the State of Colorado. Following training, study interventionists will receive up to approximately 60 min of weekly supervision or case consultation from the study investigators.

#### 3.7.2 Assessment of treatment fidelity

Treatment fidelity will be assessed by a self-report questionnaire administered to study interventionists following completion of the study session. The study team elected not to record study sessions for fidelity monitoring due to the concerns regarding participant reactions to recordings in the setting of a trial focused on PTSD (characterized in part by hyperarousal, paranoia) and the sensitivities associated with discussing firearm storage practices.

### 3.8 Safety protocol

The study will be monitored by a Data and Safety Monitoring Board (DSMB). The DSMB includes four individuals with expertise spanning suicide prevention, firearm safety, PTSD, clinical trials methodology, and biostatistics. The DSMB will meet after the first 20 to 30 patients have been enrolled in the study, with subsequent meetings occurring every six months. If the DSMB or the investigator team determines that the risks of participating outweigh any potential benefits, the study may be discontinued.

Because all study procedures will take place virtually, including the intervention being delivered over the HIPAA-compliant Zoom platform, study staff will ensure that they have the participant's current location at the beginning of the consent visit and the intervention visit. Study staff will monitor for adverse events (AEs) and serious adverse events (SAEs), which will be reported to the IRB and DSMB according to standard practices. This trial is not specifically recruiting individuals with current or past suicidal symptoms (e.g., suicidal ideation, suicide attempts). However, as noted, all participants will complete the CRP intervention. We will follow standard clinical procedures if a participant were to report suicidal symptoms necessitating intervention (e.g., emergency rescue). We will also provide participants with a resource guide that lists information for national and local emergency services, crisis hotlines, a national map for out-of-home firearm storage and locations for purchase of locking devices.

### 3.9 Data collection and analysis

#### 3.9.1 Statistical analysis for primary outcome

The primary endpoint will be analyzed using a generalized linear mixed model (GLMM) with a logit link with a random intercept per participant (Hypothesis 1a). The actual firearm storage practice score at the 6-month follow-up assessment is a continuous value from 0 to 4. The primary outcome is the improvement from baseline, which is a binary transformation of the score. It measures whether the difference between the 6-month and baseline scores is >0. If the score difference exceeds 0, the transformed primary outcome is 1 (and 0 otherwise). We will model the primary outcome (improvement at 6 months) using a GLMM with a logit link, incorporating longitudinal information from all follow-up time points. This model accounts for the missingness of measurements at follow-up time points. Similar to the 6-month score, we will include dichotomized scores from post-intervention, 1-month, and 3-month measures in the GLMM with a random intercept representing each participant. The treatment effect of PSG-T will be compared between the two arms, with terms for intervention arm, baseline firearm storage practice scores, interaction between intervention and the follow-up time points, interaction between baseline score and follow-up time, and other covariates specified *a priori*. This model formulation was found to have the least amount of bias when baseline measures were subtracted from follow-up measures and patients had differential missingness of follow-up measures between intervention and control groups [45]. There are no interim analyses planned.

#### 3.9.2 Statistical analysis for secondary outcomes

Secondary endpoints include evaluating the efficacy of PSG-T compared with PSG on knowledge about the link between firearm storage practices and suicide risk and intentions to store firearms securely (Hypothesis 1b and 1c). We will use GLMM with an appropriate link function and a random intercept per participant to model the between- and within-group differences of outcomes over time [46]. For secondary outcomes reported in Likert scales, we will conduct sensitivity analysis by treating them as ordinal outcomes in the cumulative logit mixed model. To prevent model convergence issues in the cumulative logit model, we will assess the distribution of the ordinal by each category's relative proportions and pre-specify whether we will analyze the raw scale or merged category scores. We will use causal mediation analysis to evaluate PTSD-related negative cognitions about the world as a potential mechanism of change in PSG-T for increasing knowledge, intentions, and behaviors regarding safe firearm storage practices (Hypothesis 2) [47].

#### 3.9.3 Model covariates, diagnostics, and interpretation

Covariates will be included in the multivariate analysis if they are associated with missingness or be determined to be adjusted for *a priori*. Continuous outcomes will be transformed if normality assumptions are violated. Goodness of fit statistics will be calculated and model fitting diagnostics will be assessed to examine influential points, outliers, and heteroscedasticity. There is a single primary endpoint, and no adjustment will be made for multiple comparisons concerning secondary endpoints and analyses; appropriate caution will therefore be used in interpreting the results of hypothesis testing for these analyses.

#### 3.9.4 Missing data

We expect minimal missingness (at most 10%) for the primary outcome at 6-month follow up, based on prior successes recruiting and retaining participants who report firearm ownership, and/or PTSD symptoms and the low missingness rate in prior related studies at 6-month follow-up [17,48]. Before the final analysis, missing data patterns will be assessed to determine if missingness is random/ignorable [49,50]. The logistic regression use in the primary analysis will use all available data and will adjust for covariates associated with missingness. Multiple imputation with chained equations for imputing outcomes missing at random will be considered. Patten-mixture models or sensitivity analysis will be considered if outcomes are not missing at random.

#### 3.9.5 Power

This study is powered according to the standardized effect size, Cohen's h, of the primary endpoint without considering correlations between longitudinal measures, which will provide a larger (more conservative) sample size estimate than if the study was powered based on the longitudinal correlation estimate in a GLMM [51]. Cohen's h is the standardized effect size for the between-group difference in proportions and is not affected by control group proportions [51,52]. Based on our preliminary result of the proportion of new uses of firearm locking devices in the intervention group (PSG-T, 30% = 55%–25%) and the control group (11.5% = 39%–27.5%), we calculated a Cohen's h of 0.47. A sample size of 150 (75 in each group) produces 80% power (α = 0.05) to detect a Cohen's h of 0.47 in measuring the between-group differences of proportions of reported new secure firearm storage behaviors. The proposed sample size of *N* = 168 participants (84 in each group) allows us to have >80% statistical power for the primary analysis and will yield 80% power to detect any subgroup-specific effect or standardized secondary outcomes of 0.47 or greater between PSG-T and control. The power will be at least 60% if the standardized subgroup-specific effect size is at least 0.35.

We used Monte Carlo simulation with 1000 replications to assess the power of *N* = 168 for the secondary outcomes, namely the mediation analysis of the PTSD-World in the PSG-T effect to increase knowledge, intentions, and behaviors regarding safe firearm storage practices [53]. Assuming the outcome standard deviation is 0.5, direct effect of PSG-T on PTSD is 0.6 and direct effect of PTSD on outcomes is 0.5, and the standard deviation is 1.3 for PTSD-World from Sexton et al. [54], a sample size of 168 allows us to detect a mediation effect of 0.30 (in Cohen's h scale) with 86 % power (α = 0.05).

## 4 Discussion

This manuscript provided an overview of the methodology and protocol of an RCT that will examine the efficacy of PSG-T compared with PSG among firearm-owning adults who screen positive for PTSD related to a victimization trauma (e.g., physical assault, sexual assault, military combat) and who do not currently store all their firearms securely (e.g., unlocked and/or unloaded). Given that the non-secure storage of personally owned firearms is associated with elevated suicide risk for the firearm owner and others in the home, and given that the existing PSG intervention has demonstrated diminished efficacy for individuals with elevated PTSD symptoms, identifying a novel, PTSD-focused LMSC intervention has the potential to reduce firearm-related harms, including suicide. If PSG-T demonstrates success in prompting secure firearm storage beliefs and practices, then PSG-T will address a critical healthcare gap for at-risk individuals with PTSD.

### 4.1 Consideration of alternative study designs

There are five primary study design considerations that warrant mention (see Table 3).

**Table 3:** Considerations of alternative study designs.

Consideration	Decision and Rationale
**Inclusion Criteria: Positive PTSD Screen vs. PTSD Diagnosis**	We elected to screen for PTSD using the PC-PTSD-5, consistent with PTSD screening recommendations in the VA/DOD CPG [55]. We considered whether to include participants with a PTSD diagnosis using the reference standard Clinician-Administered PTSD Scale for DSM-5 (CAPS-5) [56]. We decided to select individuals who screen positive for PTSD using the total PC-PTSD-5 for three reasons: (1) the PC-PTSD-5 is considerably briefer and more feasible to administer in the context of study inclusion screening (i.e., 5 vs. 60+ minutes); (2) assessing for symptoms rather than diagnoses aligns with findings from our pilot data indicating that existing LMSC have diminished efficacy for individuals with elevations on the total PCL-5 score [57]; and (3) this approach will align with clinical operations within the DOD, Veterans Health Administration, and other healthcare systems that typically rely on screening instruments such as the PC-PTSD-5 to make treatment-planning decisions.
**Inclusion Criteria: PTSD Symptoms Related to a Victimization Trauma**	We are limiting the sample to individuals who screen positive for PTSD related to their experience of a victimization trauma—that is, those that are interpersonal in nature, such as physical assault, sexual assault, or combat. Individuals who experience other traumatic events, such as natural disasters (e.g., hurricanes) or motor vehicle accidents, are ineligible unless they have also experienced a victimization trauma for which they screen positive for PTSD. We considered whether to limit participation on this criterion. However, unpublished evidence from our group suggests that among individuals with a victimization trauma history (but not an accident or injury trauma history), PTSD symptoms are associated with non-secure firearm storage practices.
**Intervention: Control Condition**	We elected to use an active control (i.e., PSG) rather than an inactive or attention control (e.g., health and stress counseling as in the original PSG trial) for two primary reasons. This approach would ensure that all participants, all of whom report firearm ownership and the non-secure storage thereof, would receive at least the PSG intervention, which has demonstrated efficacy in prior trials (cf. beneficence). This approach would also provide an answer to the question: does PSG-T outperform PSG for individuals who screen positive for PTSD?
**Intervention: Inclusion of Crisis Response Planning**	We decided to include CRP. One concern was that the CRP intervention would introduce unnecessary heterogeneity into the study methodology. However, CRP is inert regarding the outcomes of interest (i.e., there is no LMSC component of CRP) and the administration of CRP is balanced across conditions. Furthermore, in many clinical care settings, LMSC would not be administered without some sort of safety planning intervention.
**Intervention: Distribution of Firearm Locking Devices**	We considered whether to provide participants with locking devices, such as cable locks. The initial PSG RCT was a 2x2 factorial design (i.e., LMSC vs. control, provision of cable locks vs, no cable locks), and findings indicated that the combination of LMSC and gun lock provision was not superior to either intervention alone [58]. This suggests that distributing cable locks does not enhance LMSC. Additionally, cable locks are the most affordable locking device and, therefore, would be an appropriate device to consider regarding future scalability. However, firearm owners cite cable locks as among the least preferable device [59] and there are knowledge gaps in proper cable lock use [60].

## 5 Summary

Firearm injury is the most common suicide method in the U.S., and the non-secure storage of firearms may be associated with elevated risk of suicide. LMSC is one recommended approach to promote secure firearm storage and, therefore, decrease the risk of firearm-related harms such as suicide. One common LMSC protocol, PSG, has demonstrated efficacy in promoting secure firearm storage, but a secondary analysis of that RCT demonstrated that PSG did not outperform control for individuals with high levels of PTSD symptoms. Our group developed an adaptation of PSG—PSG-T—for use in individuals with PTSD. This project will test the efficacy of PSG-T among firearm-owning adults who screen positive for PTSD related to a victimization trauma and who do not currently securely store all their firearms.

## CRediT authorship contribution statement

**Ian H. Stanley:** Writing – review & editing, Writing – original draft, Visualization, Project administration, Methodology, Investigation, Funding acquisition, Conceptualization. **Julia Finn:** Writing – review & editing, Writing – original draft, Project administration. **Kathleen M. Flarity:** Writing – review & editing, Methodology, Conceptualization. **Mengli Xiao:** Writing – review & editing, Writing – original draft, Methodology, Conceptualization. **Rachel L. Johnson:** Writing – review & editing, Methodology. **Jaclyn C. Kearns:** Writing – review & editing, Methodology. **Natalie L. Wilver:** Writing – review & editing, Methodology. **Steven J. Berkowitz:** Writing – review & editing, Methodology, Conceptualization. **Michael D. Anestis:** Writing – review & editing, Methodology, Conceptualization. **Marian E. Betz:** Writing – review & editing, Methodology, Conceptualization. **Joseph A. Simonetti:** Writing – review & editing, Methodology, Conceptualization.

## Disclaimer

Opinions, interpretations, conclusions, and recommendations are those of the authors and do not reflect an endorsement by or the official policy or position of the US Department of Defense, the US Government, or the authors’ employers.

## Funding statement

This work is supported by the Office of the Assistant Secretary of Defense for Health Affairs through the Congressionally Directed Medical Research Program (CDMRP) FY23 Traumatic Brain Injury Psychological Health Research Program (TBIPHRP) with an award to the University of Colorado School of Medicine (PI: Stanley; Award No. HT9425-24-1-0693). The funding source has no involvement in the collection, analysis and interpretation of data, the writing of this manuscript, or the decision to submit the manuscript for publication.

## Declaration of competing interest

The authors declare that they have no known competing financial interests or personal relationships that could have appeared to influence the work reported in this paper.

## Data Availability

No data were used for the research described in the article.
